# Tetraethyl 2,2′-(2,3,5,6-tetrafluoro-*p*-phenylenedimethylene)dipropanoate^1^

**DOI:** 10.1107/S1600536808027207

**Published:** 2008-08-30

**Authors:** Haitao Xi, Yajun Gao, Xiaoqiang Sun, Qi Meng, Yan Jiang

**Affiliations:** a School of Chemistry and Chemical Engineering, Jiangsu Polytechnic University, Changzhou 213164, People’s Republic of China

## Abstract

In the mol­ecule of the title compound, C_22_H_26_F_4_O_8_, a crystallographic inversion centre is located at the centroid of the benzene ring. C—H⋯F and C—H⋯O intra­molecular hydrogen bonds are observed as well as an inter­molecular C—H⋯O inter­action.

## Related literature

For related literature, see: Benetti *et al.* (1995 ▶); Howard *et al. *(1996 ▶); Thalladi *et al.* (1998 ▶).
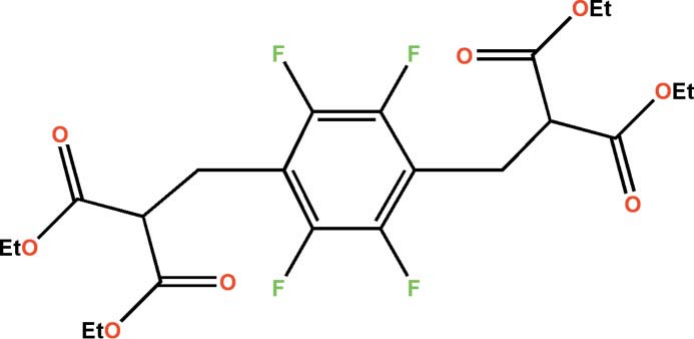


## Experimental

### 

#### Crystal data

C_22_H_26_F_4_O_8_
*M* *_r_* = 494.43
Orthorhombic, 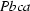

*a* = 9.833 (5) Å
*b* = 8.797 (4) Å
*c* = 27.587 (14) Å
*V* = 2386 (2) Å^3^
*Z* = 4
Mo *K*α radiation
μ = 0.12 mm^−1^
*T* = 291 (2) K
0.30 × 0.26 × 0.24 mm

#### Data collection

Bruker SMART APEX CCD diffractometer
Absorption correction: multi-scan (*SADABS*; Bruker, 2000 ▶) *T*_min_ = 0.96, *T*_max_ = 0.97
11819 measured reflections
2343 independent reflections
1411 reflections with *I* > 2σ(*I*)
*R*_int_ = 0.059

#### Refinement

*R*[*F* ^2^ > 2σ(*F* ^2^)] = 0.055
*wR*(*F* ^2^) = 0.097
*S* = 1.02
2343 reflections
156 parameters
H-atom parameters constrained
Δρ_max_ = 0.16 e Å^−3^
Δρ_min_ = −0.15 e Å^−3^

### 

Data collection: *SMART* (Bruker, 2000 ▶); cell refinement: *SAINT* (Bruker, 2000 ▶); data reduction: *SAINT*; program(s) used to solve structure: *SHELXTL* (Sheldrick, 2008 ▶); program(s) used to refine structure: *SHELXTL*; molecular graphics: *SHELXTL*; software used to prepare material for publication: *SHELXTL*.

## Supplementary Material

Crystal structure: contains datablocks 271217a, I. DOI: 10.1107/S1600536808027207/gw2045sup1.cif

Structure factors: contains datablocks I. DOI: 10.1107/S1600536808027207/gw2045Isup2.hkl

Additional supplementary materials:  crystallographic information; 3D view; checkCIF report

## Figures and Tables

**Table 1:** Hydrogen-bond geometry (Å, °)

*D*—H⋯*A*	*D*—H	H⋯*A*	*D*⋯*A*	*D*—H⋯*A*
C4—H4*A*⋯F2	0.97	2.42	2.852 (3)	107
C7—H7*A*⋯O2	0.97	2.29	2.667 (3)	102
C8—H8*C*⋯O2^i^	0.96	2.54	3.472 (4)	162

Symmetry code: (i) 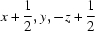.

**Fig. 1.**
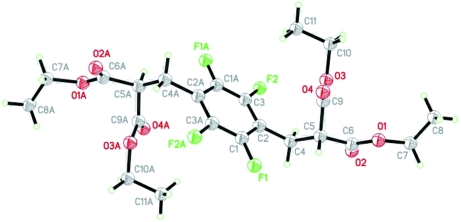
View of the molecule (I),showing the atom-labelling scheme. (thermal ellipsoids are shown at 30% probability levels).[Symmetry code:(A) -x+2, -y+1,-z]

**Fig. 2.**
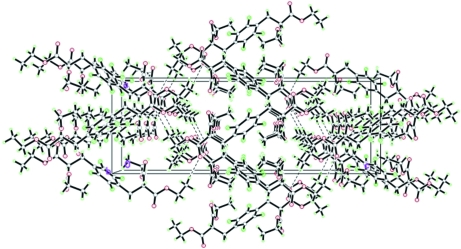
The molecular packing diagram in the crystal for (I) (Dashed lines indicate hydrogen bonds).

## supplementary crystallographic information

### Comment

β-Keto esters are multicoupling reagents with electrophilic and nuclephilic sites that have proven to be valuable tools in the synthesis of a wide variety of molecular systems(Benetti *et al.*,1995). In the present paper, we report the crystal structure of the title compound, (I). The molecule of (I) lies on a crystallographic inversion center located at the middle of the benzene ring. Selected bond distances and angles are given in Table 1. One pair of symmetrically related ethyl groups was found to be disordered over two orientations (Fig.1) The feature of the title compound in packing is based on C-H···O intramolecular interaction and C-H···F intramolecular interaction which had been reported in related references (Howard *et al.*, 1996; Thalladi *et al.*, 1998)(Fig.2); the C4-H4a···F2 distance is 2.852 Å. the C7-H7a···O2 distance is 2.667 Å and the C8-H8C···O2 distance is 3.472 Å.

### Experimental

A mixture of 1,4-bis(bromomethyl)-2,3,5,6-tetrafluorobenzene (1.67g,5mmol), ethyl malonate(1.53mL,10mmol),potassuium carbonate(1.38g 10mmol) and acetonitrile(25mL) was stirred and refluxed for 8h. The solvent was evaporated on a rotary evaporator and the resulting oil was chromatographed on a silica-gel column,yielding the title compound (1.78g,69%). Crystals appropriate for data collection were obtained by slow evaporation of an acetonitrile solution at 283K. (m.p. 324-326K).

### Crystal data

**Table:**

C_22_H_26_F_4_O_8_	*D*_x_ = 1.376 Mg m^−^^3^
*M**_r_* = 494.43	Melting point = 324–326 K
Orthorhombic, *P**b**c**a*	Mo *K*α radiation λ = 0.71073 Å
Hall symbol: -P 2ac 2ab	Cell parameters from 3968 reflections
*a* = 9.833 (5) Å	θ = 5.5–26.8º
*b* = 8.797 (4) Å	µ = 0.12 mm^−^^1^
*c* = 27.587 (14) Å	*T* = 291 (2) K
*V* = 2386 (2) Å^3^	Block, colorless
*Z* = 4	0.30 × 0.26 × 0.24 mm
*F*_000_ = 1032	

### Data collection

**Table:**

Bruker SMART Apex CCD diffractometer	2343 independent reflections
Radiation source: sealed tube	1411 reflections with *I* > 2σ(*I*)
Monochromator: graphite	*R*_int_ = 0.059
*T* = 291(2) K	θ_max_ = 26.0º
phi and ω scans	θ_min_ = 2.5º
Absorption correction: multi-scan(SADABS; Bruker, 2000)	*h* = −11→12
*T*_min_ = 0.96, *T*_max_ = 0.97	*k* = −10→5
11819 measured reflections	*l* = −34→34

### Refinement

**Table:**

Refinement on *F*^2^	Secondary atom site location: difference Fourier map
Least-squares matrix: full	Hydrogen site location: inferred from neighbouring sites
*R*[*F*^2^ > 2σ(*F*^2^)] = 0.055	H-atom parameters constrained
*wR*(*F*^2^) = 0.097	*w* = 1/[σ^2^(*F*_o_^2^) + (0.03*P*)^2^ + 0.22*P*] where *P* = (*F*_o_^2^ + 2*F*_c_^2^)/3
*S* = 1.02	(Δ/σ)_max_ < 0.001
2343 reflections	Δρ_max_ = 0.16 e Å^−^^3^
156 parameters	Δρ_min_ = −0.15 e Å^−^^3^
Primary atom site location: structure-invariant direct methods	Extinction correction: none

### Special details

**Table:**

Geometry. All esds (except the esd in the dihedral angle between two l.s. planes) are estimated using the full covariance matrix. The cell esds are taken into account individually in the estimation of esds in distances, angles and torsion angles; correlations between esds in cell parameters are only used when they are defined by crystal symmetry. An approximate (isotropic) treatment of cell esds is used for estimating esds involving l.s. planes.
Refinement. Refinement of F^2^ against ALL reflections. The weighted R-factor wR and goodness of fit S are based on F^2^, conventional R-factors R are based on F, with F set to zero for negative F^2^. The threshold expression of F^2^ > 2sigma(F^2^) is used only for calculating R-factors(gt) etc. and is not relevant to the choice of reflections for refinement. R-factors based on F^2^ are statistically about twice as large as those based on F, and R- factors based on ALL data will be even larger.

### Fractional atomic coordinates and isotropic or equivalent isotropic displacement parameters (Å^2^)

**Table:**

	*x*	*y*	*z*	*U*_iso_*/*U*_eq_	
C1	0.9879 (2)	0.6145 (3)	0.03274 (8)	0.0390 (6)	
C2	0.9112 (2)	0.4839 (3)	0.04088 (8)	0.0355 (5)	
C3	0.9249 (2)	0.3719 (3)	0.00603 (8)	0.0389 (6)	
C4	0.8172 (2)	0.4682 (3)	0.08408 (8)	0.0454 (7)	
H4A	0.7622	0.3774	0.0803	0.054*	
H4B	0.7565	0.5550	0.0853	0.054*	
C5	0.8979 (2)	0.4582 (3)	0.13216 (8)	0.0419 (6)	
H5	0.9305	0.5599	0.1409	0.050*	
C6	0.8013 (3)	0.4001 (3)	0.17246 (8)	0.0499 (7)	
C7	0.7966 (3)	0.3238 (3)	0.25401 (9)	0.0570 (7)	
H7A	0.7203	0.2646	0.2422	0.068*	
H7B	0.7619	0.4028	0.2752	0.068*	
C8	0.8912 (3)	0.2252 (3)	0.28077 (11)	0.0633 (8)	
H8A	0.9243	0.1468	0.2596	0.095*	
H8B	0.8448	0.1799	0.3078	0.095*	
H8C	0.9664	0.2846	0.2924	0.095*	
C9	1.0178 (2)	0.3515 (3)	0.12940 (8)	0.0448 (7)	
C10	1.0781 (3)	0.0853 (3)	0.11522 (9)	0.0538 (7)	
H10A	1.0372	−0.0112	0.1239	0.065*	
H10B	1.1521	0.1054	0.1376	0.065*	
C11	1.1313 (3)	0.0783 (3)	0.06437 (9)	0.0568 (7)	
H11A	1.0590	0.0505	0.0427	0.085*	
H11B	1.2026	0.0039	0.0625	0.085*	
H11C	1.1666	0.1760	0.0553	0.085*	
F1	0.98045 (14)	0.72947 (17)	0.06482 (5)	0.0522 (4)	
F2	0.85239 (14)	0.24185 (19)	0.01115 (5)	0.0546 (4)	
O1	0.87007 (16)	0.3924 (2)	0.21311 (5)	0.0495 (5)	
O2	0.68696 (19)	0.3633 (2)	0.16648 (7)	0.0587 (5)	
O3	0.97511 (17)	0.2082 (2)	0.11861 (6)	0.0545 (5)	
O4	1.13167 (17)	0.3861 (2)	0.13604 (6)	0.0526 (5)	

### Atomic displacement parameters (Å^2^)

**Table:**

	*U*^11^	*U*^22^	*U*^33^	*U*^12^	*U*^13^	*U*^23^
C1	0.0430 (13)	0.0427 (15)	0.0313 (11)	0.0002 (12)	−0.0017 (10)	−0.0024 (11)
C2	0.0358 (12)	0.0442 (14)	0.0266 (10)	0.0010 (11)	0.0017 (9)	0.0088 (11)
C3	0.0432 (13)	0.0455 (15)	0.0280 (11)	−0.0024 (13)	−0.0063 (10)	0.0057 (11)
C4	0.0443 (13)	0.0598 (18)	0.0322 (11)	0.0021 (13)	0.0061 (11)	0.0094 (12)
C5	0.0462 (14)	0.0537 (17)	0.0258 (10)	−0.0021 (13)	0.0034 (10)	0.0042 (11)
C6	0.0569 (17)	0.0594 (19)	0.0335 (12)	0.0139 (15)	0.0119 (12)	0.0100 (13)
C7	0.0707 (18)	0.0593 (18)	0.0411 (13)	0.0087 (15)	0.0088 (13)	0.0088 (14)
C8	0.0652 (19)	0.0536 (18)	0.0712 (19)	0.0072 (16)	0.0121 (15)	0.0161 (16)
C9	0.0354 (14)	0.071 (2)	0.0284 (12)	−0.0039 (13)	−0.0018 (10)	0.0072 (13)
C10	0.0566 (16)	0.0553 (18)	0.0494 (14)	0.0151 (15)	0.0027 (13)	0.0066 (14)
C11	0.0589 (17)	0.0521 (16)	0.0594 (16)	0.0145 (14)	0.0128 (14)	−0.0038 (15)
F1	0.0598 (9)	0.0513 (9)	0.0457 (7)	−0.0051 (8)	0.0158 (7)	−0.0146 (7)
F2	0.0615 (9)	0.0584 (10)	0.0439 (8)	−0.0110 (8)	0.0038 (7)	0.0030 (8)
O1	0.0582 (10)	0.0578 (12)	0.0324 (8)	0.0049 (9)	0.0083 (8)	0.0063 (8)
O2	0.0526 (11)	0.0613 (14)	0.0621 (12)	−0.0012 (10)	0.0191 (9)	0.0198 (10)
O3	0.0466 (10)	0.0574 (13)	0.0596 (11)	0.0119 (10)	−0.0048 (9)	−0.0020 (10)
O4	0.0399 (10)	0.0566 (12)	0.0612 (11)	−0.0093 (9)	−0.0100 (8)	−0.0039 (10)

### Geometric parameters (Å, °)

**Table:**

C1—F1	1.346 (3)	C7—C8	1.471 (3)
C1—C3^i^	1.376 (3)	C7—H7A	0.9700
C1—C2	1.393 (3)	C7—H7B	0.9700
C2—C3	1.383 (3)	C8—H8A	0.9600
C2—C4	1.514 (3)	C8—H8B	0.9600
C3—F2	1.355 (3)	C8—H8C	0.9600
C3—C1^i^	1.376 (3)	C9—O4	1.174 (3)
C4—C5	1.548 (3)	C9—O3	1.361 (3)
C4—H4A	0.9700	C10—O3	1.484 (3)
C4—H4B	0.9700	C10—C11	1.499 (3)
C5—C9	1.509 (3)	C10—H10A	0.9700
C5—C6	1.549 (3)	C10—H10B	0.9700
C5—H5	0.9800	C11—H11A	0.9600
C6—O2	1.182 (3)	C11—H11B	0.9600
C6—O1	1.311 (3)	C11—H11C	0.9600
C7—O1	1.469 (3)		
			
F1—C1—C3^i^	118.6 (2)	O1—C7—H7B	110.0
F1—C1—C2	119.0 (2)	C8—C7—H7B	110.0
C3^i^—C1—C2	122.3 (2)	H7A—C7—H7B	108.4
C3—C2—C1	115.0 (2)	C7—C8—H8A	109.5
C3—C2—C4	122.8 (2)	C7—C8—H8B	109.5
C1—C2—C4	122.2 (2)	H8A—C8—H8B	109.5
F2—C3—C1^i^	118.8 (2)	C7—C8—H8C	109.5
F2—C3—C2	118.5 (2)	H8A—C8—H8C	109.5
C1^i^—C3—C2	122.6 (2)	H8B—C8—H8C	109.5
C2—C4—C5	111.52 (19)	O4—C9—O3	124.7 (3)
C2—C4—H4A	109.3	O4—C9—C5	125.1 (3)
C5—C4—H4A	109.3	O3—C9—C5	110.2 (2)
C2—C4—H4B	109.3	O3—C10—C11	109.1 (2)
C5—C4—H4B	109.3	O3—C10—H10A	109.9
H4A—C4—H4B	108.0	C11—C10—H10A	109.9
C9—C5—C4	113.1 (2)	O3—C10—H10B	109.9
C9—C5—C6	108.1 (2)	C11—C10—H10B	109.9
C4—C5—C6	108.6 (2)	H10A—C10—H10B	108.3
C9—C5—H5	109.0	C10—C11—H11A	109.5
C4—C5—H5	109.0	C10—C11—H11B	109.5
C6—C5—H5	109.0	H11A—C11—H11B	109.5
O2—C6—O1	126.6 (2)	C10—C11—H11C	109.5
O2—C6—C5	125.0 (2)	H11A—C11—H11C	109.5
O1—C6—C5	108.3 (2)	H11B—C11—H11C	109.5
O1—C7—C8	108.5 (2)	C6—O1—C7	115.1 (2)
O1—C7—H7A	110.0	C9—O3—C10	118.5 (2)
C8—C7—H7A	110.0		

Symmetry codes: (i) −*x*+2, −*y*+1, −*z*.

### Hydrogen-bond geometry (Å, °)

**Table:**

*D*—H···*A*	*D*—H	H···*A*	*D*···*A*	*D*—H···*A*
C4—H4A···F2	0.97	2.42	2.852 (3)	107
C7—H7A···O2	0.97	2.29	2.667 (3)	102
C8—H8C···O2^ii^	0.96	2.54	3.472 (4)	162

Symmetry codes: (ii) *x*+1/2, *y*, −*z*+1/2.
